# An innovative hippocampal-sparing whole-brain radiotherapy planning approach via the Halcyon system: achieving lower hippocampal doses

**DOI:** 10.3389/fonc.2025.1652684

**Published:** 2025-09-02

**Authors:** Ran Tang, Maoying Lan, Xingru Sun, Yue Luo, Anyan Gu, Qing Liu, Yingjing Li, Guozi Yang, Zhenyu Pan, Guanhua Deng

**Affiliations:** ^1^ Department of Radiation Oncology, The Affiliated Huizhou Hospital, Guangzhou Medical University, Huizhou, China; ^2^ School of Public Health, Nanchang University, Nanchang, Jiangxi, China; ^3^ Department of Radiation Oncology, The First Affiliated Hospital of Guangzhou Medical University, Guangzhou, China; ^4^ Department of Radiotherapy, Southern Medical University Hospital of Integrated Traditional Chinese and Western Medicine, Guangzhou, China; ^5^ People’s Hospital of Shaodong, Shaoyang, China; ^6^ Nanjing Tongrentang Chinese Medicine, Huizhou, China; ^7^ Department of Radiation Oncology, Guangdong Provincial People’s Hospital Guangdong Academy of Medical Sciences), Southern Medical University, Guangzhou, China

**Keywords:** Halcyon, hippocampus, whole brain radiotherapy, volumetric modulated arc therapy, treatment planning

## Abstract

**Objective:**

To evaluate the feasibility and dosimetric benefits of Halcyon-based coplanar dual-arc volumetric modulated arc therapy (VMAT) for hippocampal-avoidance whole brain radiotherapy (HA-WBRT).

**Methods:**

Twenty-one HA-WBRT patients were replanned using dual-arc VMAT (collimator 23°/293°) on Halcyon and Truebeam. The planning target volume (PTV) was segmented into three substructures and optimized with different weight parameters. Dosimetric parameters of PTV, monitor units (MUs), does to organs-at-risk(OARs), hippocampal normal tissue complication probability (NTCP) and gamma passing rate were recorded.

**Results:**

All plans met RTOG 0933 criteria. The Halcyon plans demonstrated significantly better homogeneity index (HI) and V_30Gy_ of the PTV (HI: 0.105 *vs.* 0.121, *P*<0.001; V_30Gy_: 97.1% *vs.* 96.3%, *P*<0.001), alongside reduced hippocampal dose (D_100%_: 626.8 *vs.* 695.0cGy; D_mean_: 850.0 *vs.* 898.4cGy; D_max_: 1348.1 *vs.* 1399.8 cGy; NTCP: 34.16% *vs.* 31.67%, *P ≤* 0.001), OARs sparing improved for Lens D_max_ (495.0 *vs.* 525.8cGy, *P* = 0.001), Optic nerves D_max_(3047.7 *vs.* 3077.6cGy, *P* = 0.006), and eyes D_mean_(927.1 *vs.* 937.9cGy, *P* = 0.009). The average gamma passing rates were higher for Halcyon than Truebeam (3%/2mm: 99.96% *vs.* 99.85; 2%/2mm: 99.83% *vs.* 99.49%).

**Conclusions:**

Under the innovative planning approach, redefined hippocampal-sparing radiotherapy using Halcyon system, providing superior prescription dose coverage, improved OAR sparing, and reduced hippocampal NTCP.

## Introduction

The incidence of brain metastases has been steadily increasing in recent years (1, 2). Despite advances in systemic therapies, the efficacy of chemotherapy in controlling brain metastases remains limited due to the restrictive nature of the blood-brain barrier (3). Whole-brain radiotherapy (WBRT) has demonstrated efficacy in improving local control and extending overall survival in patients with brain metastases (4). However, the neurotoxic effects of WBRT on the central nervous system have become a growing concern (5). Studies have demonstrated that radiation-induced hippocampal damage significantly impairs neurocognitive functions, particularly those related to learning, memory, and spatial processing (6). A multicenter phase II clinical trial (RTOG 0933) revealed that hippocampal-avoidance WBRT (HA-WBRT) effectively preserves patients’ neurocognitive functions and improves their quality of life (7).

Over the past decades, HA-WBRT have been developed to use intensity modulated radiation therapy (IMRT), volumetric modulated arc therapy (VMAT), and tomotherapy (TOMO) techniques as listed in **Table 1** (8–22). Gondi et al. (8) found that TOMO technology offers more advantages in HA-WBRT. However, the high cost of TOMO equipment makes it unaffordable for many small-scale hospitals. Wang et al. (9) have reported that HA-WBRT based IMRT techniques takes a long time for patients on the couch, which may cause patients discomposure. Dosimetric performance of conventional VMAT for HA-WBRT has been reported in previous studies following RTOG 0933 criteria, suggesting that VMAT irradiations use non-coplanar multi-arc irradiation. However, non-coplanar VMAT techniques increase the risk of tumor movement and extend treatment time.

**Table 1:** Summary of HA-WBRT in the literature.

Ref.	Prescription	Linac and tech.	Plan quality	Hippocampus
Krayenbuehl J, et al. (2017) (10).	30Gy/10F	Trilogy Linac (Varian) 4Arcs_VMAT (2Arcs coplanar and 2Arcs non-coplanar)	V_100%_=92% HI=0.24	D_100%_= 8.1Gy D_mean_=7.3Gy D_max_=14.1Gy
Wang S, et al. (2017) (11).	30Gy/10F	Truebeam (Varian) 2 Arcs_VMAT coplanar	V_100%_=91.49% HI=0.28 CI=0.84	D_100%_=9.275Gy D_max_=16.0Gy
Gondi V, et al. (2010) (8).	30Gy/10F	Varian linac IMRT Non-coplanar	V_100%_=93% HI=0.3	D_max_=15.3Gy
Nevelsky A, et al. (2013) (12).	30Gy/10F	Infinity (Elekta) IMRT Non-coplanar	V_100%_=92% HI=0.36	D_100%_=8.37Gy D_max_=14.35Gy
Yokoyama K, et al. (2022) (13).	30Gy/10F	Halcyon 2–4 Arcs_VMAT Coplanar	V_100%_=95% HI=0.19	D_50%_=7.89Gy D_max_=14.32Gy
		Tomotherapy	V_100%_=95% HI=0.24	D_50%_=8.02Gy D_max_=12.63Gy
Xue J, et al. (2023) (14).	30Gy/10F	Axesse (Elekta) 2 Arcs_VMAT Non-coplanar	V_100%_=95% HI=0.249 CI=0.821	D_100%_=8.03Gy D_mean_=11.71Gy D_max_=16.81Gy
Zhang HW, et al. (2024) (15).	30Gy/10F	Truebeam 2 Arcs_VMAT Coplanar	V_100%_=95% HI=1.1 CI=0.84	D_mean_=11.77Gy D_max_=17.13Gy
		Tomotherapy	V_100%_=95% HI=1.05 CI=0.88	D_mean_=9.23Gy D_max_=15.42Gy
Yuen AHL, et al. (2020) (16).	30Gy/10F	Truebeam 4 Split-arcs partial_VMAT Coplanar	V_100%_=94.79% HI=0.23	D_100%_=7.86Gy D_mean_=9.16Gy D_max_=13.23Gy
Yuen AHL, et al. (2022) (17).	30Gy/10F	Truebeam 4 Split-arc partial_VMAT+2 static fields Coplanar	V_100%_=94.69% HI=0.24	D_100%_=7.92Gy D_mean_=9.21Gy D_max_=13.31Gy
Li MH, et al. (2022) (18).	30Gy/10F	Tomotherapy	V_100%_=96.56% HI=0.07 CI=0.815	D_mean_=10.7Gy D_max_=15.5Gy
		Synergy 4 Arcs_VMAT Coplanar	V_100%_=92.95% HI=0.219 CI=0.823	D_mean_=11.2Gy D_max_=15.2Gy
Takaoka T, et al. (2021) (19).	30Gy/10F	Tomotherapy	D_95%_=29.9Gy HI=0.259 CI=1.30	D_100%_=9.3Gy D_mean_=11.1Gy D_max_=14.7Gy
Wang BH, et al. (2015) (9)	30Gy/10F	Varian IX 2 Arcs_VMAT Coplanar	V_95%_=95% HI=0.13 CI=0.88	D_median_=10.30Gy D_max_=13.92Gy
Fu Q, et al. (2021) (20).	25Gy/10F	VersaHD (Elekta) 4 Arcs_VMAT	V_100%_=91.2% HI=0.084 CI=0.839	D_mean_=6.35Gy D_max_=7.90Gy
NCT01780675 Trial (21)	25Gy/10F	–	V_100%_>95% D_98%_>25Gy D_2%_<37.5Gy	D_mean_<8.5Gy
NRG CC001 Trial (22)	30Gy/10F	–	V_100%_>95% D_98%_>25Gy D_2%_<37.5Gy	D_100%_<9Gy D_max_<16Gy

In recent years, the Halcyon has gained widespread adoption in clinical practice due to its innovative design features. Unlike conventional C-arm accelerators, the Halcyon employs a circular ring gantry structure, eliminating the need for fixed jaws and enabling a ring rotation speed of 24°/s/eedi times that of C-arm LINACs. Additionally, the Halcyon is equipped with a dual-layer multi-leaf collimator (MLC) featuring 29 proximal and 28 distal leaves, which effectively minimizes leakage and transmission. The Halcyon exclusively utilizes a 6 MV flattening filter-free (FFF) photon beam, further enhancing its efficiency and precision in delivering high-quality radiotherapy. To the best of our knowledge, only a few studies have been reports of HA-WBRT using Halcyon. The results from Yokoyama et al. (13) demonstrated that three-arc Halcyon treatment plan was effective in handling hippocampus sparing whole-brain radiotherapy. However, the three-arc design prolonged prolong treatment time and increase costs. Here, we propose a novel coplanar dual-arc VMAT technique on the Halcyon platform that incorporates both target segmentation and collimator angle optimization, and systematically evaluate the dosimetric characteristics of HA-WBRT using coplanar dual-arc VMAT on Halcyon and Truebeam platforms.

## Materials and methods

### Patient selection

We retrospectively studied twenty-one patients who underwent HA-WBRT from June 2024 to December 2024. The cohort consisted of six males and four females, with a median age of 49 years (range: 33 – 70 years). All patients were diagnosed with non-hematologic malignancies confirmed through histopathological or cytological examination, with magnetic resonance imaging (MRI) showing brain metastases located at least 5 mm away from the hippocampus. Local approval was granted, and written informed consent was obtained.

### Simulation

Patients were immobilized in the supine position using a thermoplastic mask. CT images were acquired using a Brilliance Big Bore CT scanner (Philips, Netherlands) with a slice thickness of 2.5 mm, covering the region from the scalp to the upper edge of the second cervical vertebra. Additionally, contrast-enhanced T1-weighted MRI scans with a slice thickness of 1 mm were performed within two weeks before radiotherapy. CT and MRI images were fused in the Eclipse v16.1 treatment planning system to facilitate precise hippocampal delineation by radiation oncologists.

### Target and organs at risk delineation

Following the Radiation Therapy Oncology Group (RTOG) atlas, the hippocampus was manually delineated using fused CT and contrast-enhanced T1-weighted MRI images. A 5-mm three-dimensional margin around the hippocampus was designated as the hippocampal avoidance region (HA). The clinical target volume (CTV) was defined as the whole brain excluding the HA region. The planning target volume (PTV) was created by expanding the CTV by 3 mm while excluding the HA region. The prescription dose for the PTV was 30Gy in 10 fractions, with at least 95% of the PTV volume receiving the prescribed dose. Dose constraints for the PTV, hippocampus, and other organs at risk (OARs) are listed in **Table 2**.

**Table 2:** Dosimetric compliance criteria for hippocampal avoidance.

Parameter	Dose constraints		
PTV	V_30Gy_≥95%	D_2%_≤37.5Gy	D_98%_≥25Gy
Hippocampus	D_max_ ≤ 16Gy	D_100%_≤9Gy	
Optic nerves	D_max_ ≤ 33Gy		
Lens	D_max_ ≤ 7Gy		

### Equipment parameters

The Halcyon designed with a ring gantry from Varian Corporation in the United States was employed, equipped with dual-layer MLCs (29 proximal and 28 distal leaves with a 5-mm resolution) and a 6 MV FFF photon beam with a maximum dose rate of 800 MU/min. For comparison, the Truebeam linac featured a single-layer MLC with 60 leaves (40 central leaves at 5-mm width and 20 peripheral leaves at 10-mm width), a dynamic jaw tracking system, and a maximum dose rate of 1400MU/min. All treatment plans were designed using the Eclipse v16.1 treatment planning system.

### Plan design

**Figure 1:**
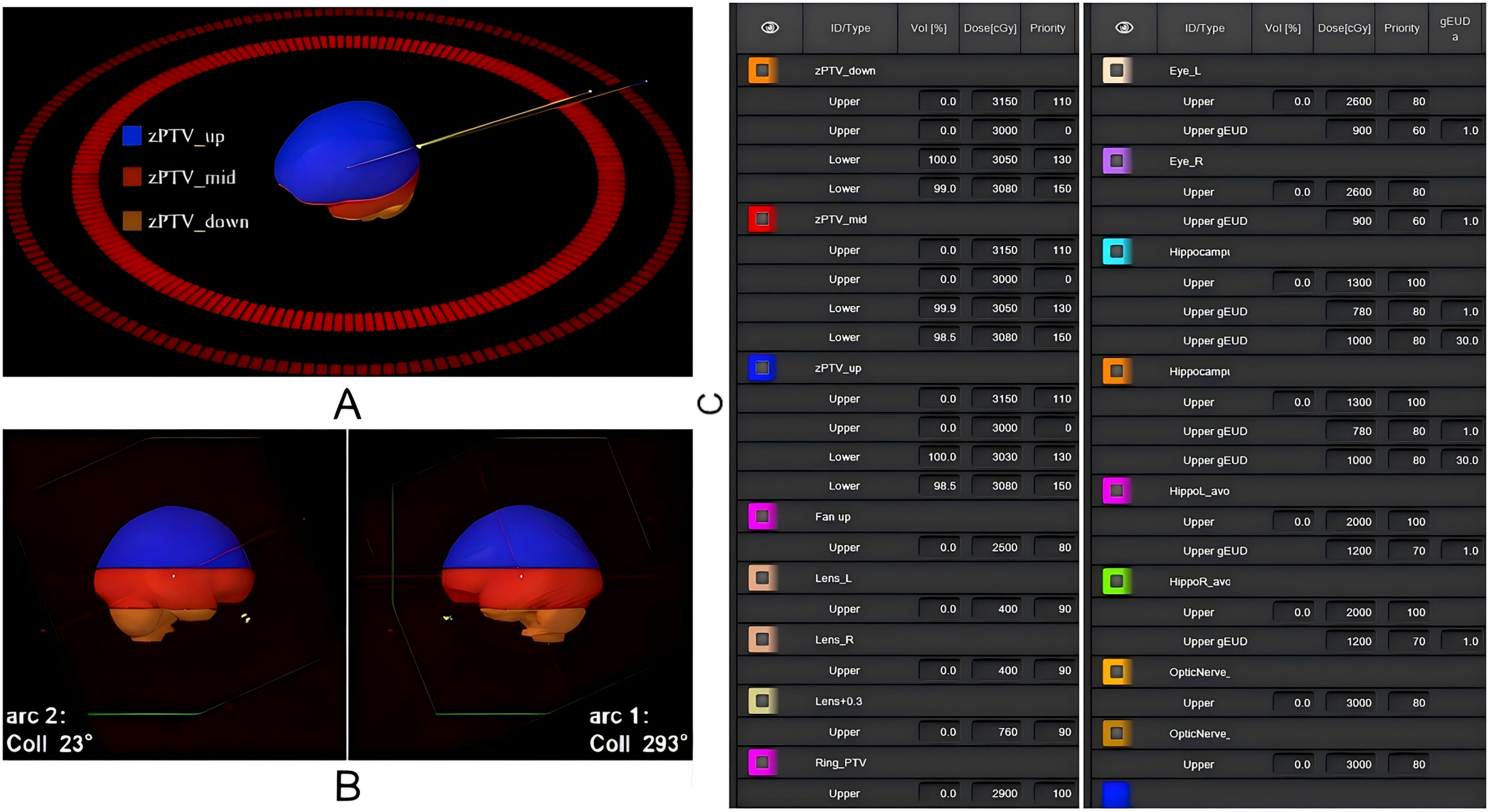
Schematic representation of the radiotherapy plan design and key target structure optimization parameters. **(A)** Logical segmentation of the PTV structure; **(B)** Gantry angle settings for dual-arc VMAT; **(C)** Key optimization parameters for target structures in the treatment plan.

To optimize PTV coverage while sparing the hippocampus, the PTV was segmented into three sub-structures: zPTV_up (from the upper PTV boundary to the superior edge of the HA region), zPTV_mid (the PTV portion overlapping the HA region), and zPTV_down (from the inferior edge of the HA region to the lower PTV boundary). This segmentation strategy enhanced modulation efficiency during treatment planning optimization (**Figure 1A**).

As illustrated in **Figure 1B**, to ensure a consistent and unbiased comparative evaluation between the selected machine models under identical clinical conditions, all treatment plans utilized coplanar dual-arc VMAT with collimator angles set at 23° and 293°. Optimization was performed using the photon optimization (PO) algorithm, and dose calculations were conducted with the Acuros XB algorithm at a grid resolution of 2.5 mm. Identical dose constraints and optimization parameters were applied to both Halcyon and Truebeam plans to ensure comparability (**Figure 1C**).

### Plan evaluation

The plan quality was assessed using dose-volume histograms (DVHs). PTV evaluation metrics included V_30Gy_ (%), conformity index (CI), and homogeneity index (HI), calculated using the following Equation 1 (23):


(1) $$\left\{ \begin{array}{c} \text{CI}=\frac{V_{t,ref}^{2}}{{\text{V}}_{\text{t}}\times {\text{V}}_{\text{ref}}}\\ \text{HI}=\frac{D_{2\%}-D_{98\%}}{D_{50\%}} \end{array}\right.$$

In the formulas, $V_{t,ref}$ represents the volume receiving the prescription dose, is the target volume, and is the volume of the prescription dose within the target volume. $D_{2\%}$ , $D_{98\%}$ and $D_{50\%}$ represent the doses received by 2%, 98%, and 50% of the target volume, respectively. The CIcloser to 1 indicates better dose conformity to the target, while the HI closer to 0 reflects more uniform dose distribution within the target.

The evaluation parameters of OARs include D_100%_, D_mean_, and D_max_ for the hippocampus; D_max_ for the lens and optic nerves; and D_mean_ for the eyeball. Additionally, the total monitor units (MUs) for all plans were recorded.

### Normal tissue complication probability

The normal tissue complication probability (NTCP) is a quantitative measurement of the probability that a dose of radiation will have an undesirable effect on an organ. The following mechanistic of formula is used to calculate the NTCP, as shown in Equations 2, 3 (24):


(2) $$NTCP=\frac{1}{\surd 2\pi }\int_{-\infty }^{t}\text{exp}(-\frac{x^{2}}{2})dx$$

(3) $$t=\frac{EQD_{2}(D_{40})-TD_{50}}{m\cdot TD_{50}}$$

$EQD_{2}(D_{40})$ was $EQD_{2}$ received by 40% of bilateral hippocampal volume, $TD_{50}$ was the $EQD_{2}(D_{40})$ valuecorresponding to a 50% probability of neurocognitive decline, and *m* represented the slope of the dose-response curve. Moreover, $TD_{50}$ and *m* were estimated to be 14.88 Gy and 0.54 by Gondi et al (24).

Biologically equivalent dose in 2-Gy fraction ( $EQD_{2}$ ) to 40% of the bilateral hippocampi was evaluated according to the following Equation 4 (25):


(4) $$EQD_{2}=D\cdot \frac{d+\alpha /\beta }{2+\alpha /\beta }$$

Where *D* represented the total dose and *d* represented the dose per fraction. An α/β ratio for the hippocampus was assumed to be 2 (25).

### Dose verification

3D gamma passing rate analysis on dose images of all treatments was performed using the Portal Dosimetry module in Varian Eclipse. Halcyon plans were verified using its built-in digital megavolt imager, with a pixel resolution of 1280 × 1280 (0.336 mm per pixel) and an active detection area of 43 cm × 43 cm. Truebeam plans were verified using the a-Si1000 electronic portal imaging device, with a pixel resolution of 1024 × 768 (0.39 mm per pixel) and an active detection area of 40 cm × 30 cm. The gamma analysis criteria were set as follows: a dose threshold of 10%, dose tolerance/distance to agreement of 3%/2 mm and 2%/2 mm, respectively. The passing rates for all treatment plans were recorded and analyzed.

### Statistical analysis

Statistical analyses were performed using SPSS v25.0 and Origin 2022. The Shapiro-Wilk test was employed to assess data normality. Normally distributed data were expressed as mean ± standard deviation and analyzed using paired t-test, while non-normally distributed data were expressed as median (interquartile range) and analyzed using Wilcoxon test. A two-tailed α-level of 0.05 was considered statistically significant.

## Results

### Dose distribution and DVH comparison

**Figure 2:**
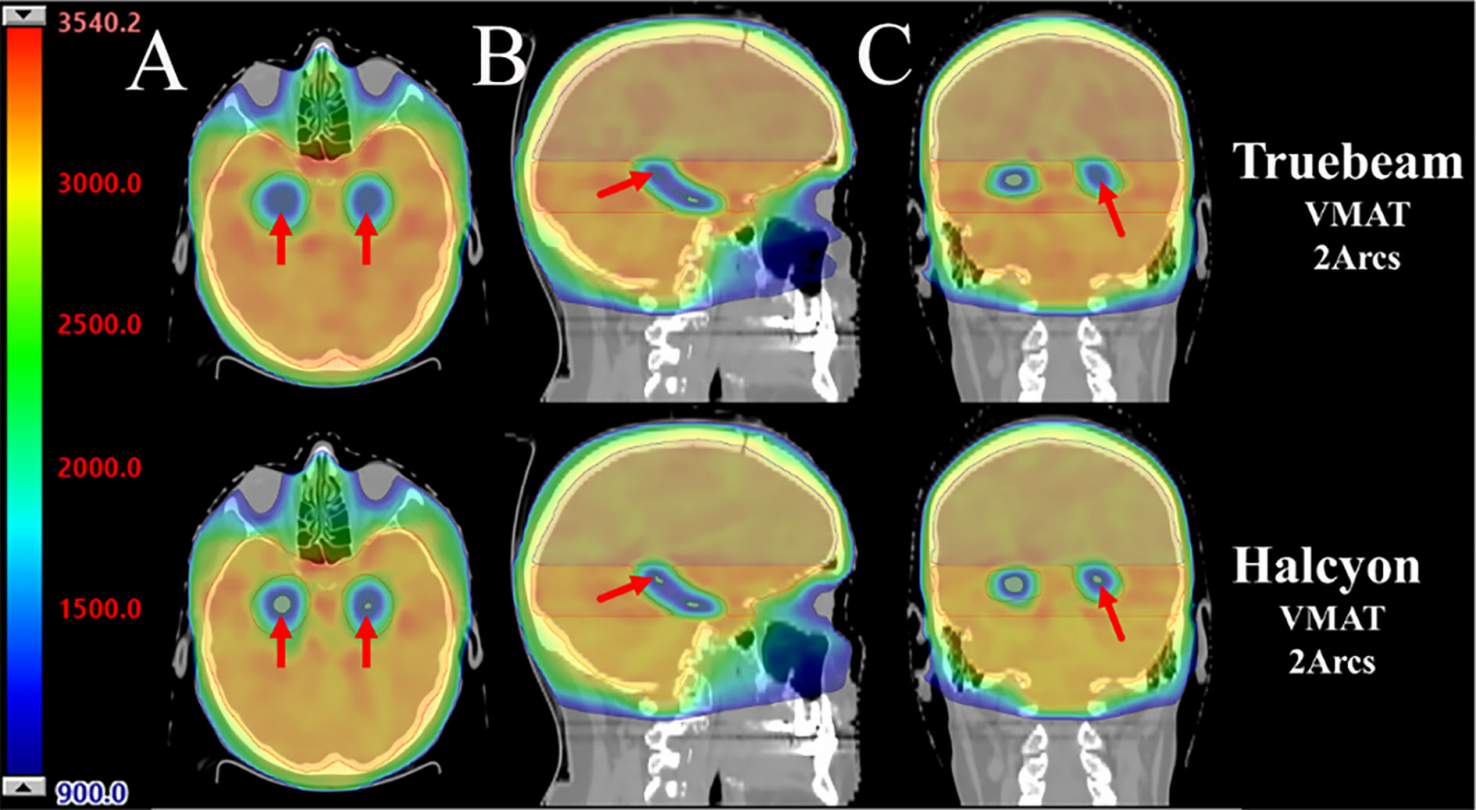
The dose distribution of Truebeam and Halcyon applying double arc coplanar VMAT for a representative patient. **(A-C)** Dose distribution at the axial, sagittal and coronal views. The top and bottom figures are the Truebeam and Halcyon plans, respectively.

**Figure 3:**
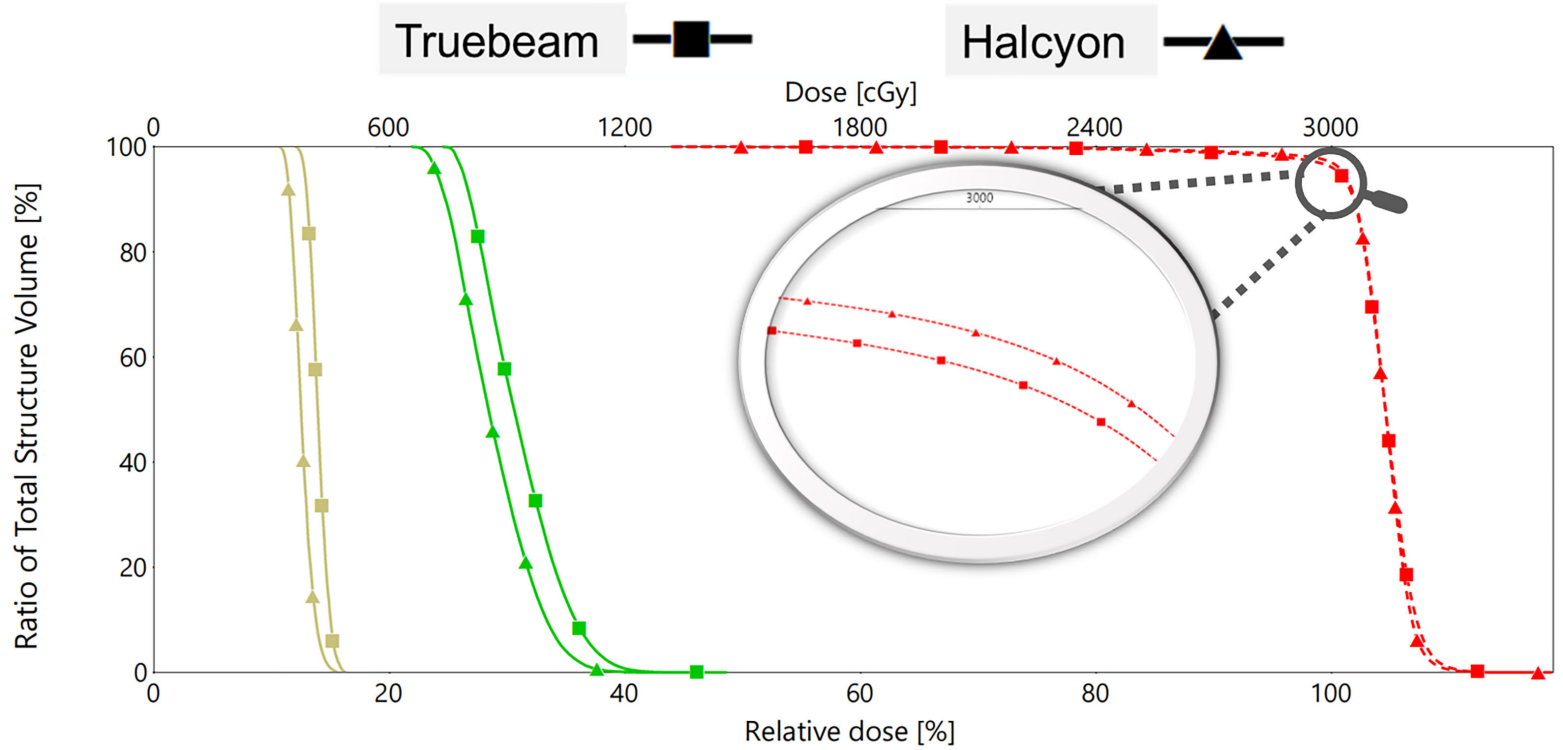
DVHs comparison for PTV and OARs between Truebeam and Halcyon applying VMAT. The yellow, green and red line represents the physical dose exposure to the lens, hippocampus and PTV, respectively.

The Halcyon plan demonstrated superior PTV coverage and hippocampal sparing compared to the Truebeam plan, as illustrated in **Figure 2**. The DVHs for the same patient, shown in **Figure 3**, indicate that both plans met clinical requirements. Notably, the Halcyon plan achieved OAR doses well below tolerance limits and demonstrated a more favorable DVH profile compared to the Truebeam plan.

### Dosimetric parameters and monitor unit comparison

All plans achieved ≥ 95% PTV coverage at the prescription dose. The Halcyon plan demonstrated superior coverage at 97.1%, compared to 96.3% for the Truebeam plan (*P*< 0.001). In terms of dose homogeneity, the Halcyon plan achieved a significantly lower median HI value than the Truebeam plan (0.105 *vs.* 0.121, *P*< 0.001). Conversely, the CI was marginally better in the Truebeam plan compared to the Halcyon plan (1.105 *vs.* 1.127, *P*< 0.001). However, the Halcyon plan required significantly more MUs than the Truebeam plan (1083.0 *vs.* 903.0, *P*< 0.001), as shown in **Table 3**.

**Table 3:** Comparison of dosimetric parameters and MUs for PTV $\left[\text{n}=21,\;\bar{\text{x}}\pm \text{s}/\text{M}(\text{Q}1,\text{Q}3)\right]$ .

Parameters	Truebeam	Halcyon	t/z value	*P* value
CI	1.105 ± 0.051	1.127 ± 0.059	-7.069	<0.001
HI	0.121 (0.113, 0.125)	0.105 (0.099, 0.108)	-4.015	<0.001
V_30Gy_/%	96.3 ± 0.3	97.1 ± 0.3	-11.118	<0.001
MU	903.0 (884.5, 918.5)	1083.0 (1053.4, 1140.1)	-4.015	<0.001

### Dosimetric comparison for OARs

**Figure 4:**
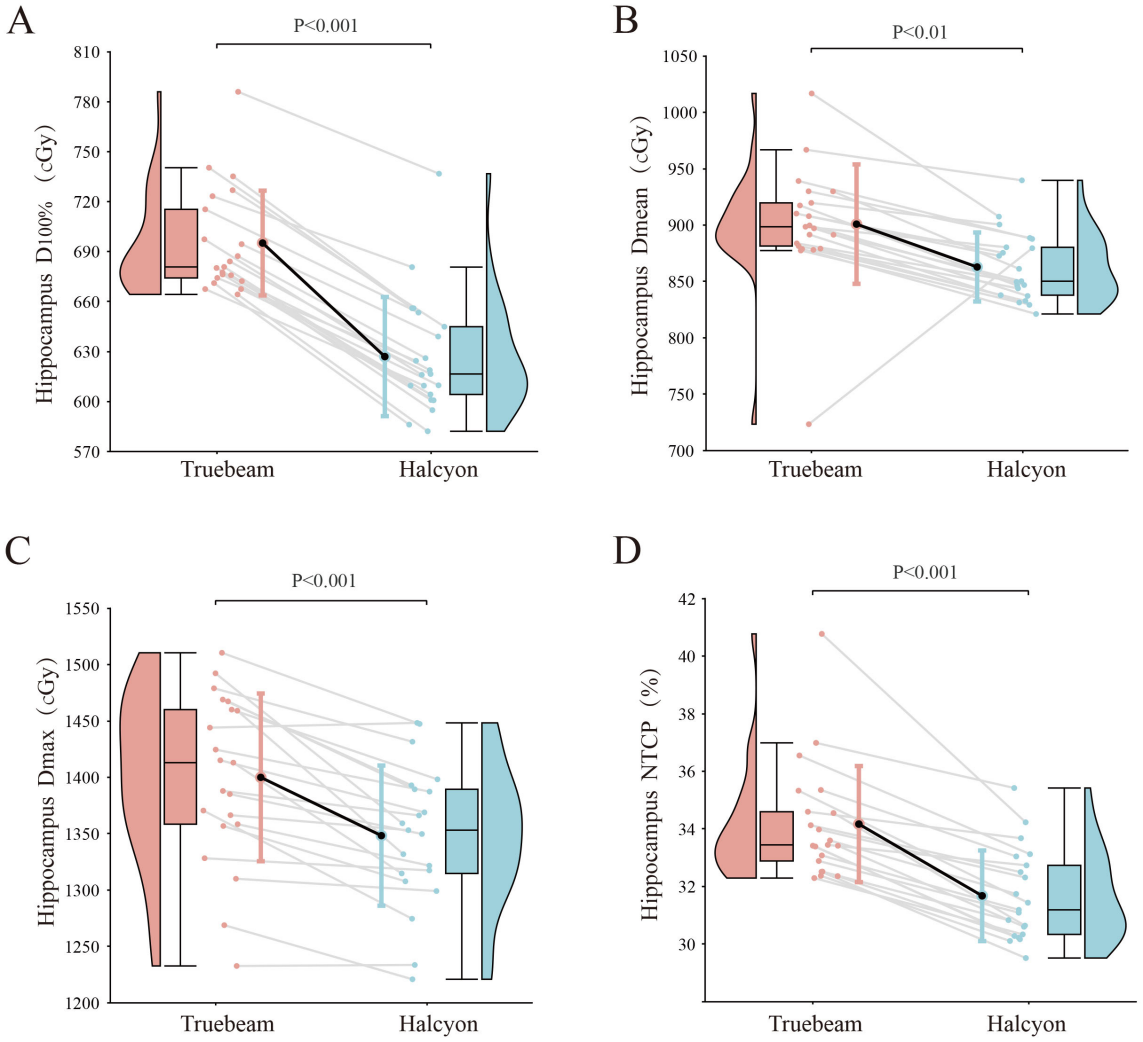
Comparison of hippocampus dosimetric parameters. The results are shown for the **(A)** D_100%_, **(B)** D_mean_, **(C)** D_max_ and **(D)** NTCP of hippocampus, respectively.

The average volume of the hippocampal avoidance region and PTV were 5.4 cm^3^ (1.5 - 8.1 cm^3^) and 1542.4 cm^3^ (1298.3 - 1872.3 cm^3^), respectively. The volume of hippocampal avoidance region was accounted for 0.35% of PTV. The Halcyon plan showed superior dosimetric performance for hippocampal protection, achieving significantly lower D_100%_, D_mean_, D_max_ and NTCP than the Truebeam plan: 626.8 ± 35.8cGy*vs.* 695.0 ± 31.5cGy (*P*<0.001), 850.0(837.4, 883.9)cGy*vs.* 898.4 (880.1, 924.7) cGy (*P* = 0.001), 1348.1 ± 62.2cGy*vs.* 1399.8 ± 74.4cGy (*P*<0.001), and 34.16 ± 2.02% vs. 31.67 ± 1.57% (p<0.001), as shown in **Figure 4**.

Additionally, the Halcyon plan achieved a significantly lower D_max_ for the lens and optic nerves and lower D_mean_ for the eyes compared to the Truebeam plan, as shown in **Table 4**.

**Table 4:** Comparison of OARs dosimetric parameters $\left[\text{n}=21,\;\bar{\text{x}}\pm \text{s}/\text{M}(\text{Q}1,\text{Q}3)\right]$ .

OARs	Parameters	Truebeam	Halcyon	t/z value	*P* value
Lens	D_max_ (cGy)	525.8 (487.9, 554.4)	495.0 (458.5, 521.3)	-3.285	0.001
Optic nerves	D_max_ (cGy)	3077.6 ± 44.7	3047.7 ± 64.7	3.080	0.006
Eyes	D_mean_ (cGy)	937.9 (916.7, 967.6)	927.1 (906.7, 947.4)	-2.624	0.009

### Gamma passing rate comparison

**Figure 5:**
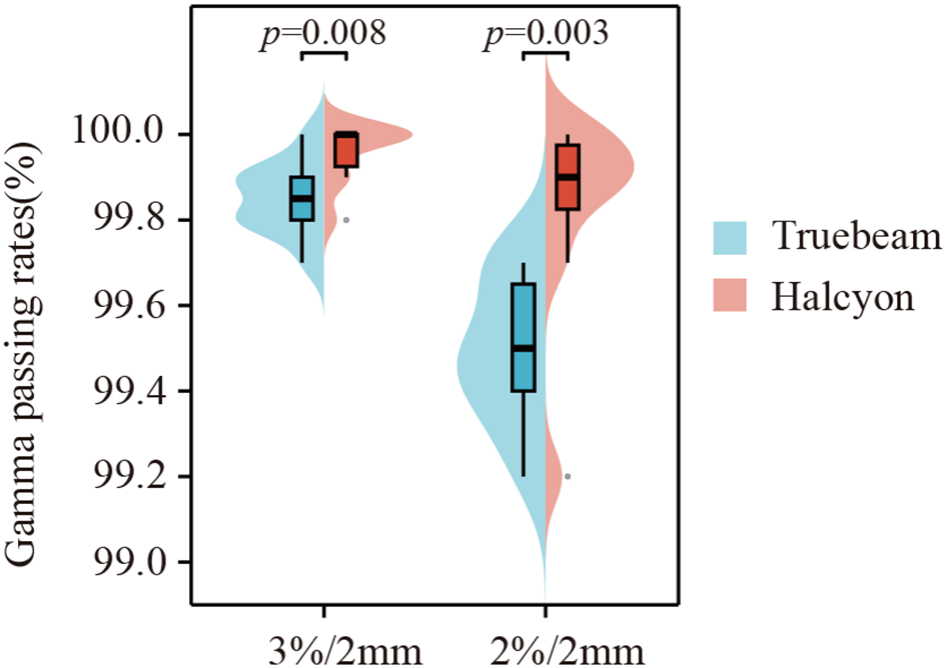
Global gamma passing rates of the VMAT plans using Truebeam and Halcyon.

All plans successfully passed the quality assurance test under the 3%/2 mm and 2%/2 mm gamma criteria, with passing rates exceeding 97%. As shown in **Figure 5**, the Halcyon plan demonstrated superior gamma passing rates compared to the Truebeam plan under both criteria: 99.96% ± 0.07% *vs.* 99.85% ± 0.08% for 3%/2 mm (z=16.5, *P* = 0.008) and 99.83% ± 0.24% *vs.* 99.49% ± 0.17% for 2%/2 mm (z=11, *P* = 0.003).

### Comparison with other studies in hippocampal-avoidance whole-brain radiotherapy

Compared to previous studies, the Halcyon plan achieved notable milestones in HA-WBRT. The Halcyon plan delivered higher prescription dose coverage (D_98%_, 29.5 Gy; D_2%_, 32.8 Gy; V_95%_, 98.7%; V_100%_, 97.1%), superior dose homogeneity (HI, 0.105), and excellent hippocampal protection (D_100%_, 6.27Gy; D_mean_, 8.50Gy; D_max_, 13.48Gy). These results are comparable or superior to outcomes reported for Tomotherapy and non-coplanar VMAT techniques.

## Discussion

HA-WBRT has been shown to be superior to standard WBRT in preserving neurocognitive function and improving patients’ quality of life (26), and it has gradually become a widely adopted therapy for brain metastases. In medical centers equipped with both conventional linear accelerators and Halcyon platforms, selecting the optimal radiotherapy device is a critical step in treatment planning. In this study, HA-WBRT plans were generated for both Halcyon and Truebeam accelerators, and differences in dose distributions for target volumes and OARs were analyzed. Both plans met the RTOG 0933 protocol and clinical requirements. The Halcyon plans demonstrated significant advantages in HI and V_30Gy_for the PTV, as well as hippocampal, lens, optic nerves, and eyes. Conversely, Truebeam plans showed a slight advantage in PTV CI, with a marginal 2.0% difference. The Halcyon plans achieved a 15.2% improvement in HI, reflecting superior dose uniformity. Although the Halcyon plans required 16.6% more MUs than Truebeam, the Halcyon’s gantry rotation speed is four times faster, significantly reducing treatment delivery time (27). It should be noted that the higher MU requirements of Halcyon systems may exacerbate several potential problems: (i) increased scatter dose, (ii) accelerated machine wear, and (iii) higher treatment costs.

In terms of hippocampal protection, the Halcyon plans demonstrated a significant reduction in D_100%_ (626.8cGy), achieving a 10.9% decrease compared to the Truebeam plans. The reductions in D_mean_ (850.0cGy *vs.* 898.4cGy) and D_max_ (1348.1cGy *vs.* 1399.8cGy) were more modest, at approximately 4.8%. While statistically significant differences were observed in plan comparisons, the clinical implications of these variations warrant further investigation. To address this issue to some extent, we employed NTCP modeling - a validated quantitative measure for assessing radiation-induced tissue damage severity. Our analysis revealed that Halcyon treatment plans demonstrated the most favorable neurocognitive protection profile, as evidenced by significantly lower NTCP values (*p*<0.001).These findings suggests that the Halcyon system offers superior protection for normal tissues surrounding by the target volume, particularly in low-dose regions. Several factors may account for this advantage:

1. Jawless Design:

The Halcyon accelerator’s jawless configuration positions the MLC leaves closer to the source. Although the leaf tips are rounded, their longer radius and straighter edges minimize the dosimetric leaf gap (DLG) to just 0.1mm (28), significantly reducing the penumbra compared to the 1.8mm DLG observed with Truebeam’s MLC design.

2. Dual-Layer MLC:

Halcyon utilizes a dual-layer, staggered MLC configuration with a transmission factor of only 0.47% (29), markedly lower than Truebeam’s average transmission of 1.5% for 6 MV beams (30). This design effectively reduces dose leakage and improves the protection of surrounding tissues.

3. Faster Leaf Motion:

The MLC leaves on the Halcyon achieve a maximum speed of 5cm/s, double Truebeam’s maximum leaf speed of 2.5cm/s. Previous studies have demonstrated that faster leaf motion enhances the sparing of OARs outside the target region (31), consistent with the findings of this study.

4. Enhanced Modulation Capabilities:

In traditional accelerators, achieving optimal modulation often requires fixing the jaw position due to the limitations of MLC movement when dealing with large target diameters and fields (32). In contrast, the Halcyon’s MLC design eliminates this restriction, allowing full extension of the leaves without carriages and enabling seamless modulation across the entire field. Truebeam, by comparison, is limited by a maximum leaf extension of 15 cm beyond the central carriage, which restricts modulation in VMAT plans for larger fields. To address these challenges, techniques such as partial arcs and smaller field sizes have been employed on Truebeam, as reported by Yuen et al. (16), achieving a target HI of 0.23 and a hippocampal D_mean_ of 9.16 Gy. However, the Halcyon platform inherently overcomes these limitations due to its innovative MLC design and superior modulation capabilities.

Rong et al. (33) compared IMRT, VMAT, and TOMO for HA-WBRT, concluding that TOMO provides superior dosimetric distribution, particularly in terms of dose uniformity. In studies conducted by Takaoka et al. (19) and Li et al. (18), TOMO achieved 95% PTV coverage with V_30Gy_, CI values of 1.3 and 0.815, and hippocampal D_max_ and D_mean_ of 14.7 Gy/11.1 Gy and 15.5 Gy/10.7 Gy, respectively. Hippocampal volume had a large effect on the planning parameters, as shown in **Table 4**. The treatment planning with the small hippocampal volume resulted in the better dose distribution of target and lower D_max_ values of hippocampus. For instance, the volume of hippocampi was 5.4 cm^3^ in our study, whereas the value was 3.95 cm^3^ described by Takaoka et al. (19). In our study, Halcyon plans demonstrated better hippocampal sparing (D_max_ of 13.48 Gy, D_mean_ of 8.50 Gy) and achieved exceptional PTV coverage and homogeneity. These findings underscore Halcyon’s competitive performance in HA-WBRT and its potential as an effective alternative to TOMO. Yokoyama et al. (13) investigated the impact of arc number (2 – 4 arcs) in Halcyon-based VMAT plans, and recommended 3 arcs for HA-WBRT, reporting a hippocampal D_max_ of 14.32 Gy. In our study, the dual-arc VMAT plan achieved a lower hippocampal D_max_ (13.48 Gy *vs.* 14.32 Gy). We attribute this improvement to two innovations in our coplanar dual-arc technique: (i) Target structure segmentation with differential weighting during optimization, enhancing the plan’s modulation capability. (ii) The orthogonal collimator angle design facilitates more conformal subfield shapes and better protection of OARs, particularly in complex spatial relationships between the target and OARs.

Non-coplanar IMRT and VMAT techniques have been explored to improve hippocampal sparing. For example, Nevelsky et al. (12) achieved hippocampal D_max_ and D_mean_ of 14.1 Gy and 7.3 Gy, respectively, using nine-field non-coplanar IMRT, though the PTV coverage (92% for V_30Gy_) was suboptimal. Subsequently, Xue et al. (14) employed a non-coplanar VMAT approach improving the V_30Gy_ coverage to 95%. and achieving a HI and CI values of 0.249 and 0.821, respectively, with hippocampal D_100%_, D_max_, and D_mean_ values of 8.03 Gy, 16.81 Gy, and 11.71 Gy. Although Halcyon does not currently support non-coplanar delivery, its HA-WBRT plan quality in our study remains competitive with these reported techniques, demonstrating comparable hippocampal sparing and robust target coverage.

It is worth noting that due to the complexity of HA-WBRT, plan quality is of paramount importance, and selecting appropriate collimator angles is a critical factor for achieving an optimal dose distribution. In this study, the collimator angles for Arc 1 (293°) and Arc 2 (23°) were set with an inter-arc angle of 90°, consistent with previous studies (34). To address the high complexity of the target structure, the zPTV_mid module, which posed greater challenges in meeting planning objectives, was assigned higher optimization weights. This segmentation and weighting strategy improved modulation efficiency during plan optimization, aligning with the modified VMAT techniques reported by Fu et al. (20). Compared to previously published data (as shown in **Table 1**), the HA-WBRT plans only using coplanar dual-arc technology in our study demonstrated superior prescription dose coverage and dose uniformity. The PTV coverage reached 97.1%, with hotspots (D_2%_) controlled within 108% of the prescription dose. In terms of hippocampal sparing, the plans achieved groundbreaking results, maintaining an average hippocampal dose below 9 Gy and reducing the lens D_max_ to less than 5 Gy.

Several limitations should be acknowledged in this study. First, this is a retrospective single-center study that lacks validation of long-term clinical outcomes. Second, although our study demonstrated favorable clinical outcomes, the small sample size (n=21) may limit the extrapolation of the results. Third, the potential impact of brain metastasis locations on plan quality was not evaluated. Future longitudinal, multicenter prospective studies will evaluate both cognitive outcomes and survival endpoints in patients with HA-WBRT.

## Conclusion

This study demonstrates that the Halcyon accelerator is a viable and efficient platform for HA-WBRT, with excellent PTV dose coverage, superior dose homogeneity, and effective hippocampal sparing while reducing treatment times. These findings provide a robust basis for further exploration and clinical adoption of the Halcyon platform in hippocampal-avoidance radiotherapy.

## Data Availability

The original contributions presented in the study are included in the article/supplementary material. Further inquiries can be directed to the corresponding author.
